# Felid Cardiopulmonary Nematodes: Dilemmas Solved and New Questions Posed

**DOI:** 10.3390/pathogens10010030

**Published:** 2021-01-02

**Authors:** Donato Traversa, Simone Morelli, Angela Di Cesare, Anastasia Diakou

**Affiliations:** 1 Faculty of Veterinary Medicine, University of Teramo, 64100 Teramo, Italy; smorelli@unite.it (S.M.); adicesare@unite.it (A.D.C.); 2 School of Veterinary Medicine, Faculty of Health Sciences, Aristotle University of Thessaloniki, 54124 Thessaloniki, Greece; diakou@vet.auth.gr

**Keywords:** cardiopulmonary nematodes, domestic cats, wildcats

## Abstract

In the past decade cardiopulmonary nematodes affecting felids have become a core research topic in small animal parasitology. In the late 2000s, an increase in studies was followed by unexpected findings in the early 2010s, which have stimulated research teams to start investigating these intriguing parasites. Prolific scientific debate and exchanges have then fostered field and laboratory studies and epizootiological surveys. New data have improved basic and applied knowledge, solved dilemmas and posed new questions. This article discusses the past and present background to felid cardiopulmonary nematodes after the last few years of intense scientific research. New data which have demonstrated the key role of *Aelurostrongylus abstrusus* and *Troglostrongylus brevior* in causing respiratory infections in domestic cats, and on the nil to negligible current importance of other species, i.e., *Troglostrongylus subcrenatus*, *Oslerus rostratus* and *Angiostrongylus chabaudi*, are presented. Biological information and hypothesized alternative routes of infection are analysed and discussed. Novel identification and taxonomical data and issues are reported and commented upon. On the whole, recent biological, ecological and epizootiological information on felid metastrongyloids is critically analysed, with the aim to answer outstanding questions, stimulate future studies, and underline new research perspectives.

## 1. A Pandora’s Box Containing Parasitic Nematodes

In 2010 the record of a practically unknown respiratory nematode in two domestic cats (*Felis catus*) from Ibiza [1] opened a Pandora’s box. Two years later, this parasite was identified in a further case in Sicily as the crenosomatid *Troglostrongylus brevior* and its role in causing verminous bronchopneumonia in domestic cats was acknowledged [2]. Until then, nematodes of the genus *Troglostrongylus* were considered affiliated to wild felids, with a limited geographic distribution and no importance in cat parasitology, whilst the angio-strongylid *Aelurostrongylus abstrusus* (the “cat lungworm”) was known as the prevailing respiratory nematode of *F. catus* [3,4]. After these first findings, *T. brevior* has been recorded in cats from more European regions, especially southern mountainous areas and islands [5,6,7,8]. These new descriptions have changed the scenario regarding metastrongyloids affecting felids, revealing dilemmas and mistakes, fostering scientific debates and posing outstanding questions [3,9,10,11].

**Figure 1:**
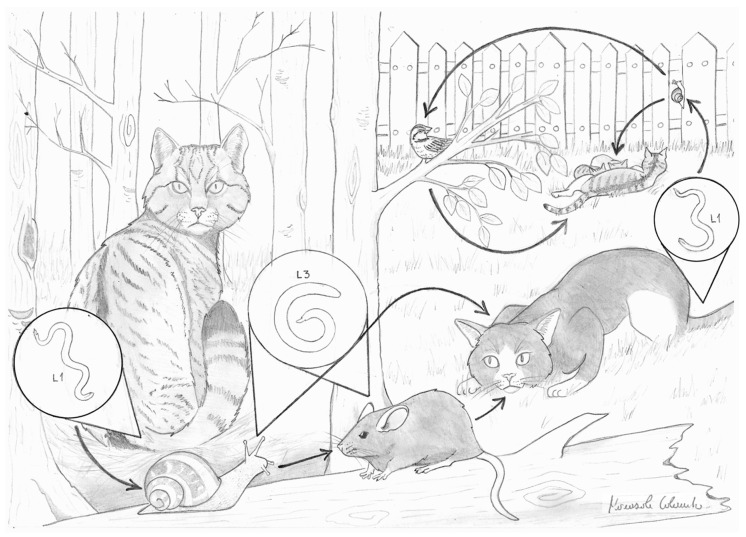
Schematic representation of the *Troglostrongylus brevior* spillover from the European wildcat (*Felis silvestris*) to the domestic cat (*Felis catus*).

**Figure 2:**
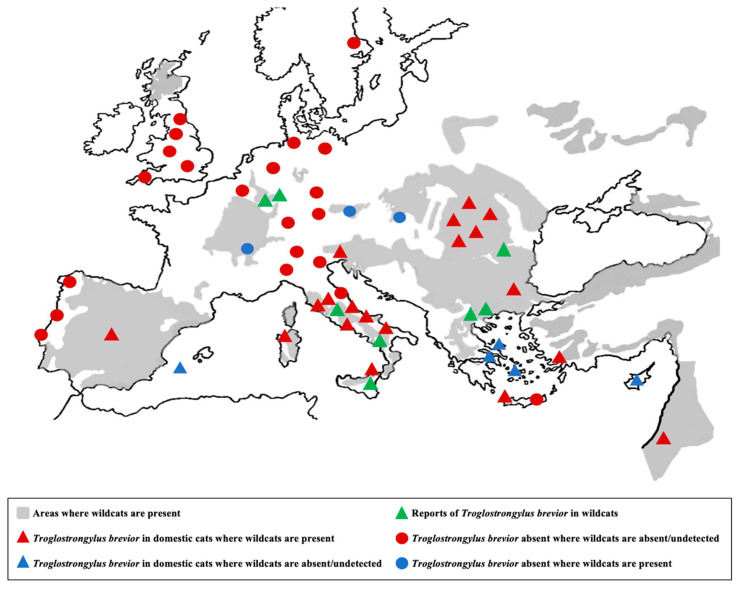
Geographic distribution of *Troglostrongylus brevior* in Europe and Middle East. The geographic distribution of *Troglostrongylus brevior* in domestic cats (red triangles) [6,7,16,17,18,19,20,21] reflects the distribution area of wildcats (in grey) [22]. *Troglostrongylus brevior* has been sporadically reported in touristic spots where wildcats are absent or undetected (blue triangles) [1,6,7]. Green triangles indicate reports of *Troglostrongylus brevior* in wildcats [23,24,25,26,27,28]; areas where the nematode has been not found in absence [29,30,31,32,33] or presence [16] of wildcats are indicated by red and blue circles, respectively.

Various speculations, investigations and hypotheses were presented aiming at understanding the origin of the unexpected records of *T. brevior* in domestic cats in the early 2010s. Evidence has indicated that under certain conditions *T. brevior* infects *F. catus* due to a spillover (Figure 1) from its natural host, i.e., the European wildcat (*Felis silvestris*). Overlapping morphological/morphometric and biological features of *A. abstrusus* and *T. brevior*, along with the sporadic occurrence of the latter, have been considered sources of frequent confusion in past identifications of lungworms [2,4,9]. According to this speculation, the emergence of feline troglostrongylosis was not “true”, but rather the result of an increased awareness, after the “reappearance” in 2010 of this neglected nematode [1,4,9]. Nevertheless, bibliographic analyses and more recent data have demonstrated that misdiagnosis can be only marginally included in an account of the emergence of feline troglostrongylosis, which rather relies on a spillover from wildcats to domestic cats. Misdiagnosis in the past was evidently the case only in sporadic detections. Soon after the first records, the pathogenic potential of *T. brevior* has been questioned [9]. To date, it is ascertained that clinical troglostrongylosis is often severe and fatal, especially in kittens and young cats. If *T. brevior* was frequently confused with *A. abstrusus*, severe and fatal aelurostrongylosis would have often been reported, but this has been extremely infrequent until now [5]. At present, the frequent life-threatening cases of troglostrongylosis, with evidence of vertical transmission, which has never been a concern for aelurostrongylosis, leave no room for lingering doubts [6,12,13,14]. Moreover, a large retrospective study demonstrated that from 2002 to 2013 *T. brevior* was diagnosed with extremely low frequency in cats living in areas enzootic for metastrongyloids [15]. To date, areas enzootic for *T. brevior* reflect the distribution area of *F. silvestris* and neighboring zones (Figure 2) [14,16,17], and regions from where the samples in the retrospective study mentioned above [15] were collected are encompassed in the distribution area of the European wildcat. In those areas a true increase of troglostrongylosis is glaring, as the infection may be responsible for 42% of metastrongyloid infections in domestic cats [17]. Other biological, clinical and pathological evidence [3,5,8,12] indicates that misdiagnosis and increase in awareness have had only a limited role in the current epizootiological scenario of cat troglostrongylosis, and that *T. brevior* has been overlooked by parasitologists. In any case, *A. abstrusus* remains a genuinely cat-affiliated lungworm and the only species that can be named “cat lungworm”.

Notwithstanding, the enhancement of knowledge has stimulated new doubts, revealed other gaps and posed further questions regarding *A. abstrusus* and *T. brevior* [5]. The booster effect has also led to the (re)discovery of other cardiopulmonary nematodes with the apparent ability to infect domestic cats, i.e., *Troglostrongylus subcrenatus* [2], *Oslerus rostratus* [34,35] and *Angiostrongylus chabaudi* [36,37]. Thus, their involvement in feline parasitology has become another priority for investigation. Until 5 years ago, no new information on felid metastrongyloidea was available, especially for Southern Europe, Italy in particular [5], though the general knowledge gaps were applicable to a broader geography. The aims of the present review are to critically summarize and discuss the large amount of epizootiological and biological data generated in the recent years, to present novel information and discuss new research perspectives.

## 2. Identity Dilemmas

### 2.1. The Importance of Microscopic Analysis

The identity of first stage larvae (L1, the diagnostic stage for metastrongyloid nematodes) in felid faeces has not been usually considered a topic worthy of investigation. In most cases L1 were identified as *A. abstrusus* or *Aelurostrongylus*-like and such an approach has been hypothesized to be at the basis of the misidentification of *T. brevior* as *A. abstrusus* (see previous section). Regardless of whether *T. brevior* has been misdiagnosed for a long time or not, comprehensive key features of *A. abstrusus* and *T. brevior* L1 have been provided [3,4,9,10]. Importantly, L1 length has been considered a key characteristic for larval identification, but it is now clear that this feature is not enough for a correct identification (Table 1). The hypothesis [9] that the overlap of L1 microscopic features has caused confusion between *A. abstrusus* and *T. brevior* was, among other reasons, based on a Table published in 2013 [3], which delineated the wide differences and discrepancies existing in the range of L1 lengths in the available literature. Accordingly, for the sake of clarity, the length of these L1 was established within precise ranges [4,9]. Today these ranges are inconsistent and should be abandoned as a very large-scale survey [16] has proved (Table 1) that the length of *A. abstrusus* and *T. brevior* L1 may be much more variable than expected [4,9]. This also means that some identification from wild felids could be correct, though the measurements are incompatible (Table 2) if the classical microscopic keys are used [3,4,9]. These deviations indicate that larval size is not enough for identifying felid metastrongyloids and that a complete morphologic and morphometric examination of the larvae, possibly followed by a molecular characterization, is necessary [4,5,11,38].

Misidentifications of *O. rostratus* as *Troglostrongylus* spp. or *A. abstrusus* have been also regarded as plausible [3]. Recent findings in domestic cats have provided microscopic and molecular information on *O. rostratus* [16,34,35], although unresolved issues remain. The majority of diagnosed cases of oslerosis in cats originated from adult parasites retrieved at necropsies [34,35,51]. Although in some cases infections could have been missed by faecal examinations, in most recent epizootiological surveys *O. rostratus* was either not found [7,8,15,18] or recorded in low prevalence [16]. To date, although advocated [5], no further studies have been performed to (i) investigate in-depth the potential growth of metastrongyloid larvae after hatching [34], (ii) provide ultimate data on the morphometry of *O. rostratus*, and (iii) validate diagnostic DNA-based tests. However, the last microscopic and genetic information available [16,34] should render more easily the identification of this rare parasite in felid faeces.

Thus far, *T. subcrenatus* has been found only in two domestic cats, in 1961 in Malawi [52] and in 2012 in Sicily [2]. Albeit that thoughtful morphometric, molecular, and phylogenetic analyses were provided for *A. abstrusus* and *T. brevior*, neither thorough larval microscopic features nor genetic data have been generated thus far for *T. subcrenatus* [2,9]. No microscopic measurements for *T. subcrenatus* L1 are reported in the articles cited in [2], which provided L1 descriptions but no molecular or phylogenetic data. The only available morphological data on *T. subcrenatus* L1 come from the first description of the parasite in a leopard (*Panthera pardus*) in 1913 [53] and in the case report from Sicily [2]. After almost 10 years from this last case, no other records of *T. subcrenatus* have been published. Information for the identification of *T. subcrenatus* L1 is still poor and stuck in the past, thus a reliable identification is difficult until thorough microscopic and genetic data are generated, and high-quality photos of L1 are published. Therefore, it is unknown why the chance of a misdiagnosis between *A. abstrusus* and *T. subcrenatus* in cats shedding L1 has not been proposed, whilst hypothesized for *T. brevior* [9]. Other features cast a shadow over *T. subcrenatus*. Morphological differences between adult *T. subcrenatus* and *T. brevior* are practically restricted to female parasite size, with *T. subcrenatus* described as bigger than *T. brevior* [2], while male *T. subcrenatus* and *T. brevior* have overlapping features in a number of descriptions [12,23,54]. The most recent comparative analysis [2], showed only negligible differences between these two species. The lack of records of *T. subcrenatus* despite extensive studies on thousands of cats could have a simpler explanation than peculiar epizootiological and biological traits: *T. subcrenatus* could be a morphotype of *T. brevior* with a limited geographic distribution, and not a separate species. Cases of morphotypes initially construed as separate species are already known for other Strongylida nematodes [55].

### 2.2. Aelurostrongylus abstrusus and Wild Felids

A few years ago it was suggested that *F. catus* is not the only proper host of the “cat lungworm” [9]. A careful analysis [10] of the literature cited in [9] has indicated that this is not the case, because none of these papers contained definite evidence (Table 2). Records in wild felids from South America [50] and other reports [44,46,47,49] are also questionable (Table 2). Nonetheless, recent morphological and genetic data have shown the unequivocal capability of *A. abstrusus* to infect wild felids. The species most often infected is the European wildcat, as shown by epizootiological surveys that revealed ~20%–60% prevalence, and single clinical or post-mortem records from Italy, Greece and Bosnia and Herzegovina [24,25,26,56]. Furthermore, a study from South Africa has reported *A. abstrusus* in caracals (*Caracal caracal*), lions and serval (*Leptailurus serval*) by microscopic and/or genetic analyses [57].

Therefore, *A. abstrusus* may infect wild felids, especially European wildcats which live in sympatry with domestic cats. Molecular and phylogenetic analyses of an informative mitochondrial DNA region have indicated that the same populations of *A. abstrusus* and *T. brevior* circulate in domestic and wild hosts from the same regions and among countries under the same routes of transmission [58]. The rare occurrence of *A. abstrusus* in large felids [57] vs. its high frequency in *F. silvestris* could rely on the different animals preyed on by these felids. For example, lions hunt animals weighing 190–550 kg [59], not typical paratenic hosts of *A. abstrusus*. Conversely, the European wildcat is frequently exposed to *A. abstrusus*, as rodents and birds are the staple of the diet of *F. silvestris* [22]. In general, *A. abstrusus* infections in wildlife occur under occasional epizootiological pressure, and the high prevalence in *F. silvestris* populations in some areas may rely on the same factors that have spurred the infections of *F. catus* with *T. brevior* [5].

### 2.3. Felid Angiostrongylosis

Records of nematodes in the pulmonary arteries of felids fit the “spillover scenario”. In the last decade *A. chabaudi* has been found in wildcats from Germany [27], Greece [24,38], Romania [60], Bulgaria [61] and Bosnia and Herzegovina [56], and in domestic cats from Sardinia and the Apennine region of Italy [5,25]. *Angiostrongylus vasorum* (a nematode of canids) has also been found in a domestic cat in Switzerland [62]. Doubt about the identity of the nematodes found in wildcats in Germany [27] were resolved by microscopic and molecular comparisons with parasites found in domestic and wildcats in various countries [24,25,36,37,38,56,60], demonstrating that the parasites found in Germany were *A. chabaudi* and not *Angiostrongylus gubernaculatus*, which remains a rare parasite of wild mustelids and canids in North America. These data proved that wildcats in Europe may be infected by *A. chabaudi*, but the clinical and epizootiological importance of the rare findings of immature *A. chabaudi* and *A. vasorum* in cats from Italy and Switzerland is likely limited to isolated cases [5,25,62]. Although *A. chabaudi* completes its lifecycle in *F. silvestris*, i.e., its natural host [38], to date it is known that *A. chabaudi* and *A. vasorum* do not reach adulthood in domestic cats [5,62]. The negligible importance of angiostrongylosis in domestic cats has also been confirmed by two recent, retrospective [19] and serological and molecular [63] studies. Thus, at the moment, (i) there is no concurrence for *Angiostrongylus* spp. in the identity dilemmas for L1 in the faeces of *F. catus*, (ii) *A. chabaudi* is not a concern for domestic cats, and (iii) these parasites are not involved in the differential diagnosis of cardiorespiratory diseases in domestic cats [5].

### 2.4. The Future

Knowledge of the identity of cardiopulmonary nematodes in wild felids is still meagre. Parasitological studies of elusive felids are difficult due to the inherent hindrances in collecting suitable samples, but further studies are encouraged to implement knowledge of their cardiopulmonary parasitoses. As a recent example, additional examinations and microscopic descriptions of parasites from kodkods (*Leopardus guigna*) in Chile would have allowed identification of *Troglostrongylus* sp. and *Angiostrongylus* sp. at the species level [64].

## 3. Felids and the Ecology of Cardiopulmonary Nematodes

The role of European wildcats in the epizootiology of *T. brevior* and its transmission to domestic cats is decisive. Domestic cats acquire troglostrongylosis almost exclusively in areas where the European wildcat is present (Figure 2), thus it is correctly considered the natural host of this parasite [25,26,28]. Therefore, spillover of troglotrongylosis from wildcats to domestic cats in areas of sympatry is a fact, despite a few descriptions from European islands where wildcats are absent. Different reasons based on the characteristics of these geographic spots may explain this phenomenon.

Indeed, *T. brevior* has been retrieved in four islands where the European wildcat is absent (Figure 1). These islands are Ibiza, where a case of troglostrongylosis in domestic cat was recorded in 2010 [1], two islands of the Aegean Sea, i.e., Mykonos and Skopelos [7], and Cyprus [8].

**Figure 3:**
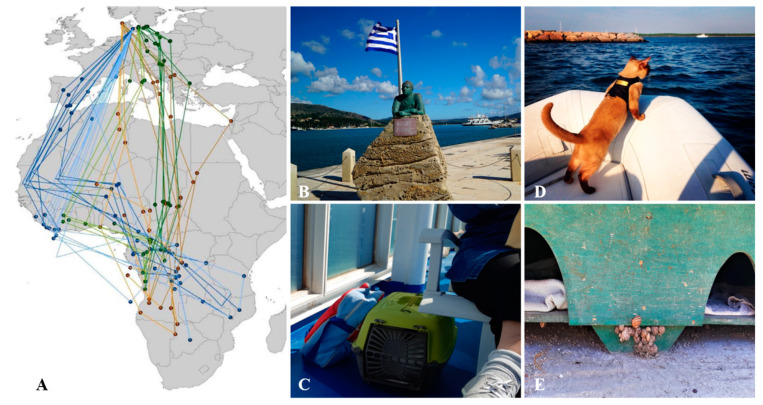
Migration, travels and islands suitability. (**A**) Complete annual migration routes, indicated in different colors, of bird species between Europe and Africa. Part of Figure 2 from [65] under the terms of the Creative Commons Attribution 4.0 International License [66]; (**B**) Statue of the famous Greek poet Nikos Kavadias (1910-1975), Argostoli harbour, Kefalonia, Greece (courtesy of Anna Votsi [67]); (**C**) Tourist travelling with her cat on a ferry boat; (**D**) Cat on a boat travelling with its owners on holiday and moving between touristic spots (courtesy of Instagram @miss_rigby_boatkitty); (**E**) Snails in a cat feeding station in Mykonos Island, Greece.

The presence of the parasite in islands is not paradoxical. It is possible that in such areas *T. brevior* has spread to domestic cats in prehistoric ages, before the contemporary coastlines were established. It is however more realistic that *T. brevior* was introduced in some way and then became enzootic. Infectious larval stages harbored by paratenic hosts (e.g., rodents, birds) could have arrived to the islands anthropogenically (e.g., with ships for trade and movements of goods) or with migrating birds (Figure 3A), that are abundant on Mediterranean islands [65]. It is equally plausible that *T. brevior* has been introduced by cats. It was a tradition for sailors to keep cats as pets in their journeys, as described by the renowned Greek poet, writer and sailor Nikos Kavvadias (Figure 3B). This tradition still continues as islands are very popular touristic spots and many pet owners travel for holidays on ferry boats with their companion animals, including cats (Figure 3C,D). Regardless of how *T. brevior* has been introduced, it has found suitable local conditions to perpetuate its biological cycle (Figure 3E).

Wildcats living in regions with high prevalence of lungworms [7,17] may display high infection prevalence and/or mixed infections by *T. brevior* and *A. abstrusus* [25,26]. Conversely, the bridging infection between domestic cats and wildlife observed for *T. brevior* and *A. abstrusus* does not occur for *O. rostratus*, *T. subcrenatus* and *A. chabaudi*.

*Oslerus rostratus* completes its life cycle in domestic cats [34], yet its affiliation to wild or domestic felids is not easy to understand. Sporadic findings, i.e., its high prevalence in cats in Majorca Island [51] and two single cases in Italy reported in a short period of time [34,35], have, however, required alertness to clarify its importance to cat parasitology. The intensification of epizootiological surveys has subsequently proved that the occurrence of *O. rostratus* is from nil to negligible in both domestic cats and wildcats even in areas where other metastrongyloids are enzootic [16,17,18,24,25,26,28,56]. Thus, *O. rostratus* remains an occasional parasite for domestic cats, and wildcats do not exert an important epizootiological pressure in areas of sympatry.

*Troglostrongylus subcrenatus* has so far been considered a parasite of large felids that live in wild and non-anthropized environments [53,68]. Importantly, epizootiological surveys and clinical or post-mortem cases published since 2012 in wildcats and domestic cats from Europe, for a total than more 3000 animals [7,15,16,18], have not revealed any additional record of *T. subcrenatus*. The taxonomic identity of *T. subcrenatus* is questionable (see Section 2.1), but if considered a valid species it has no relevance to cat parasitology and European wildlife has no role in its epizootiology. If considered as a separate taxon, the origin of the infection of the single cat from Sicily [2] is also mysterious and the absence of genetic data on these isolates prevents any further consideration (see Section 2).

Recent reports of unfertilized/immature *A. chabaudi* in single or mixed infection with *A. abstrusus* and *T. brevior* in domestic cats from Italy [36,37] have rendered mandatory a higher level of vigilance and additional investigations. Several cases of *A. chabaudi* have been reported in wildcats [24,25,26,38,56,60,61], thus it would have been reasonable to expect that *F. silvestris* is a source of infection to domestic cats (as for *T. brevior*). In fact, *A. chabaudi*, *T. brevior* and *A. abstrusus* share biological, geographical and ecological niches where intermediate and definitive hosts live in sympatry [24,37,56,69]. However, this is not the case, as serological and molecular studies conducted in the same regions have shown that domestic cats are not infected by *A. chabaudi* [19,63]. Thus, bridging infections between domestic cats and the European wildcat are observed only for *T. brevior* and *A. abstrusus*.

## 4. Novel Biological Acquisitions: Hypotheses and Facts

### 4.1. Vertical Transmission

Case reports and surveys have shown that troglostrongylosis affects mostly kittens and young cats, [70] and that a vertical transmission of *T. brevior* is beyond doubt [6,8,12,13,71,72].

Recently, based on the age of three infected kittens from the same litter and the onset of clinical signs, it has been proposed that the transmission from the queen to the litter plausibly happens via colostrum and/or milk [12]. Another publication has corroborated this hypothesis, suggesting a transmammary transmission in one-month-old kittens nursed by a queen which was shedding larvae, whilst their mother was negative at the copromicroscopic examination. Lactogenic transmission was suggested also for kittens born from the same infected queen that scored positive for *T. brevior* L1 at 18-days-old [72]. Although the infection in kittens of a few days old possibly contributes to clarify the modality of vertical transmission [12], and the relation between infected and non-infected queens living in a garden of a parasitologist and nursing their own or other kittens is a lucky coincidence offering interesting scientific observations [72], missing information prevents definitive conclusions to be drawn. No data is available on larval shedding before 30-35 days of age in the three kittens described in [12]. The one-month old kittens reported in [72] could have been infected in a different way, e.g., by intermediate and paratenic hosts reported in the same environment or by their mother, despite negative showing at the faecal examination. In this latter case, a false negative result cannot be ruled out and the lack of L1 in the faeces does not exclude a transplacental infection, that might take place when the pregnant cat ingests L3 and passes L4 to the foetuses, without patency in the queen. A transplacental transmission cannot be ultimately excluded either in the 18-days-old kittens [72], as the prepatent period for *T. brevior* reported in the literature is 24-28 days [54,73] and recent experimental data indicate that L1 are shed the at earliest 20 days after infection, when cats get infected with L3 (Raue K. and Strube C., Institute for Parasitology, Center for Infectious Diseases, University of Veterinary Medicine Hannover, personal communication). Regardless if the parasite is transmitted in utero and/or lactogenically, it is unknown whether the vertical transmission takes place after recent infection in a late phase of pregnancy (as for the roundworm *Toxocara cati* [74]), or in already infected queens that become pregnant.

### 4.2. Transmission Modes and Alternative Routes of Infection

Transmission patterns and sources of infection via intermediate and paratenic hosts have also been investigated. An experimental study in the snail *Cornu aspersum* provided data on the anatomical location of *A. abstrusus* L3 and on the influence of temperature on their development [75]. Additional information was generated on the biology (e.g., overwintering capacity) of *A. abstrusus* and *T. brevior* in *C. aspersum* experimentally infected with both nematodes [76]. It has been experimentally shown that *T. brevior* increases its development rate inside hibernated gastropods [77]. Molluscs were predicted to be intermediate hosts of *A. chabaudi* in accordance with the biology of the *Angiostrongylus* genus [37,38]. As expected, an experimental study showed that *A. chabaudi* reaches the L3 stage in the snail *C. aspersum* [78].

Usually, felids become infected by cardiopulmonary nematodes when preying on paratenic hosts like rodents, reptiles, and birds, rather than by ingesting gastropods [11,54]. A recent study has confirmed old data on the role of mice as paratenic hosts of *A. abstrusus*, indicating that L3 localize in skeletal muscles, brain, and internal organs and that they are infective to cats [79]. It has been experimentally shown that cockroaches (*Periplaneta americana*) may maintain *A. abstrusus* L3 alive in their tissues for several days [80]. Based on an old study indicating that infective L3 of the rat lungworm *Angiostrongylus cantonensis* can be released in the field via the mucus of infected intermediate hosts [81], a direct L3 ingestion from the environment was suggested as a potential route of infection [82]. Larvae of *A. abstrusus* and *T. brevior* may be released by *C. aspersum* in the mucus when snails are alive and submitted, or not, to laboratory stimuli (e.g., stirring), and when killed by submersion in water [82]. Spontaneous shedding of L3 in the faeces of experimentally infected slugs has been documented for *A. abstrusus* and some canine metastrongyloids [83].

**Figure 4:**
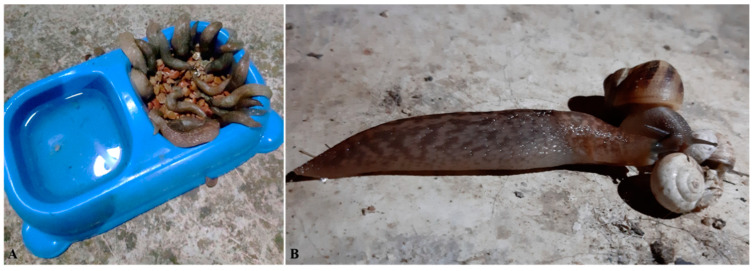
Hypothesized phenomena contributing to the biology of felid cardiopulmonary metastrongyloids. An experiment suggested that metastrongyloid third stage larvae (L3) may be released in the environment [83], but it has never been demonstrated if this happens in natural conditions. If so, infected molluscs drowned in a cat bowl or mingling in cat food (**A**) may be a potential source of infection. Another laboratory study proposed the “intermediesis”, i.e., the snail-to-snail transmission of L3 [84], which is not known whether it occurs in the field. Regardless, it would be worth investigating if similar phenomena, e.g., gastropod cannibalism and interspecific predation (**B**), may also occur.

On the whole, the aforementioned data, mostly obtained in laboratory conditions, have improved knowledge of felid cardiopulmonary nematodes. Nonetheless, laboratory settings are not identical to natural conditions in terms of characteristics of the habitat, environmental temperatures, infection rates and synchronicity (e.g., high number of L1 infecting the gastropods at once), thus the factual role of such events requires further studies in natural conditions. For example, although events that trigger L3 release may occur (Figure 4A), the likelihood and frequency of L3 release in natural conditions and the capability of these larvae to infect paratenic hosts and/or cats has never been demonstrated. This applies also to other recent acquisitions as, for example, the snail-to-snail transmission of L3 (“intermediesis”) under experimental conditions [84]. Furthermore, it would be interesting to investigate if cannibalism and interspecific predation (Figure 4B), a common behaviour of gastropods [85], could play any role in passing metastrongyloid larvae from one individual to one, or multiple others, and what is (if any) the significance of this event in the epizootiology of cardiopulmonary parasitoses. Similarly, the tendency of cats to ingest cockroaches and their role in the lifecycle of *A. abstrusus* in the field [80] are unknown. Moreover, further investigations are necessary to demonstrate if the ability of *T. brevior* L1 to develop to L3 at hibernating conditions implies a true seasonality adapted to a vertical transmission in field conditions, as hypothesized [77]. Thus, rigorous field investigations are necessary to clarify the factual significance and relevance of observations and hypotheses, as epizootiological or lifecycle data derived from natural settings have higher relevance than experimental findings. In this regard, surveys have shown that L3 are harboured by various wild-caught gastropods [69,86,87,88], i.e., widespread species that in many cases have been introduced in regions further than their native geography. However, the presence of L3 in molluscs collected in the field does not necessarily demonstrate a primary role as intermediate hosts of given species, and the mere sharing of common ecological niches does not always result in spillover or bridging infections. As an example, although some molluscs (e.g., *Helix lucorum* and *Massylaea vermiculata*) may harbour L3 of *A. chabaudi* and *A. abstrusus* [69], the vertebrate host is decisive for the perpetuation of the life cycle of a parasite, as discussed in the previous section for *A. chabaudi* and domestic cats.

### 4.3. News on Angiostrongylosis

Until 2016, *A. chabaudi* L1 had been undescribed, giving space to possible misidentifications in domestic cats as hypothesized for *A. abstrusus* and *T. brevior*. Other puzzling aspects were the intermediate hosts involved in its life cycle and the pathogenicity of the parasite to the definitive hosts [5]. The scientific cornerstone was the patent case of angiostrongylosis in a European wildcat in Greece, which demonstrated for the first time that this animal acts as definitive host of *A. chabaudi* [38]. In this report, the description of the L1 revealed species-specific characteristics enabling discrimination from other metastrongyloid larvae that can be found in felid faeces [38]. These findings, along with subsequent records from Eastern Europe [60,61], have ultimately proved that *A. chabaudi* reaches adulthood in the European wildcat, which represents its proper host.

## 5. Felids, Parasites and (Bio)Geography

While the distribution range of *A. abstrusus* is well-known, knowledge on the geographical occurrence of the other species is still incomplete.

Thus far, *T. brevior* has been described in domestic cats from Southern and Eastern Europe [1,2,7,16,19] and the Middle East [20,21,54]. It has been also recorded in wildcats from Germany [27], Italy [25,28], Greece [24,26], Romania [23] and in a Eurasian lynx from Bosnia and Herzegovina [89], but never in domestic cats from Central and Northern Europe (Figure 1).

The correlation between the distribution area of the European wildcat and *T. brevior* in domestic cats is evident. Wildcats are distributed across the Mediterranean basin and their presence has been documented in all countries of Eastern and Southern Europe, with the exception of some areas, including Ibiza, Malta, Cyprus and other small islands [22,90]. On the contrary, in Central and Northern Europe domestic cats and wildcats are sympatric only in limited areas, as a possible explanation of the lack of reports of *T. brevior*. Straying is another important driving factor, as the abundance of free-ranging cats in Eastern and Southern Europe and Middle East [91,92] increases contacts with wildcats, while stray cats are less frequent in Central/Northern European countries.

Domestic cats harbouring *A. chabaudi* have been documented only in Italy, whilst all records in wildcats derive exclusively from Southern and Eastern Europe [36,37,63]. The isolated cases recorded in Italy could be the result of an intense parasitological pressure in these areas, and/or of a higher level of vigilance. Nonetheless, given that *A. chabaudi* infections in cats are not patent and can be diagnosed only at necropsy or with purposed serological assays [63], undetected cases in other countries cannot be excluded. *Angiostrongylus felineus* has been reported only once in a jaguarundi (*Herpailurus yagouaroundi*) in Brazil [93], thus its geographical and host ranges are practically unknown.

To date, *T. brevior* and *A. chabaudi* have never been reported in felids from America, Oceania or Asia (with the exception of *T. brevior* in some Middle East areas). However, descriptions of larvae in wild felids from Brazil, Mozambique, Tanzania and Russia require clarification (Table 2). Nematodes identified as *Troglostrongylus* sp. and *Angiostrongylus* sp. have been recently found in kodkods from Chile but informative pictures of key morphological features of adult nematodes were not provided, and no description of L1 or molecular analysis were performed [64].

It is unknown whether *T. brevior* and *A. chabaudi* infect felids outside Europe, and further studies based on comprehensive microscopic and genetic analysis are warranted. On the other hand, some inherent data on the distribution of *T. brevior* and *A. chabaudi* have been generated from wild-caught gastropods. *Angiostrongylus chabaudi* has not been detected in any of the gastropods collected in Argentina [86], Brazil [87], Colombia [94], or Austria [88,95], while it was found in Greece [69]. However, *T*. *brevior* was found in Colombia and Austria [88,94] as well as Greece [69]. These results are surprising, as *T. brevior* has never been found in felids from South America or Austria. Furthermore, the ribosomal ITS2 of *T. brevior* in molluscs from Colombia (GenBank Accession Number: MH780056) and Austria (GenBank Accession Number: MK675815) [88,94] were identical to each other, with a <100% nucleotide homology with all other *T. brevior* sequences deposited in the GenBank. phylogeographic studies aiming at understanding if there is any significance related to available *T. brevior* sequences, from intermediate and definitive hosts from different regions, are thus advocated. Appropriate genetic information will also assist in elucidating the status of species ranked within the genus *Troglostrongylus* and their true geographical distribution, especially for *T. subcrenatus* (see previous sections).

## 6. Concluding Remarks: Questions Answered, Doubts Solved and Open Perspectives

The amount of data generated by recent studies on felid cardiopulmonary nematodes has provided answers to outstanding questions published five years ago and has solved inherent doubts and dilemmas [5]. These data rely on new microscopic, genetic, epizootiological and biological acquisitions which have been obtained in laboratory and field studies. It is now established that domestic cats may be infected by different metastrongyloid nematodes (Table 3).

*Aelurostrongylus abstrusus* remains the primary nematode affecting the respiratory system of domestic cats, but it can infect wild felids, especially the European wildcat, under occasional epizootiological pressure [25,26,57]. As the “cat lungworm” is apparently spreading in both domestic and wild felids, a constant epizootiological surveillance is crucial, along with implemented diagnostic, therapeutic and control measures. The European wildcat has ultimately been shown to be the natural host of *T. brevior* and *A. chabaudi* [5,25,38]. There is clear evidence that *T. brevior* is now enzootic in populations of domestic cats of southern and insular Europe and that the European wildcat has played a crucial role in fostering this establishment. It has also been demonstrated that this nematode may also circulate in domestic felids where the wild reservoir is absent. There is a significant risk of introduction in non-enzootic areas (e.g., central and northern Europe) through the movement of infected cats as a consequence of increased pet travel in Europe [7] and the high adaptability of intermediate hosts to new areas [69]. The expansion described for *T. brevior* has not been documented for other felid cardiopulmonary nematodes [15,16,17,18,19,63] and this means that *T. subcrenatus*, *O. rostratus* and *A. chabaudi* have not been underestimated nor misdiagnosed in domestic cats in the past, but they are only occasional parasites of *F. catus*. Their spreading in domestic cats is unlikely to happen under the current conditions especially because European wildlife has no factual role in any transmission patterns to domestic cats. However, a constant monitoring is essential to understand whether these nematodes of minor veterinary significance will also gain importance in cat parasitology in the future, and to validate possible diagnostic and management measures.

Major identity dilemmas have been dispelled and the identity of cardiopulmonary nematodes affecting domestic cats is now definitely established. A remaining doubt is the actual existence of *T. subcrenatus* as a separate species, as arisen from the epizootiological data obtained in the past few years, where no infections by *T. subcrenatus* have been detected in thousands of felids examined across Europe from areas where metastrongyloid are enzootic. Accordingly, *T. subcrenatus* as a valid taxon is questionable, as long as it remains without any support from detailed microscopic and genetic analyses. Further studies on adult *Troglostrongyus* spp. specimens could provide a definitive evidence as to whether *T. subcrenatus* and *T. brevior* are the same species or not.

A great deal of data on the biology of these parasites have been generated and have opened new perspectives. Alternative routes of transmission pathways, i.e., spontaneous release of L3 to the environment and the snail-to-snail L3 transmission (“intermediesis”), have been proposed for *A. abstrusus* and *T. brevior* [82,83,84]. However, the likelihood and frequency of these events in natural conditions, and the capability of L3 to infect paratenic hosts and/or cats, has never been demonstrated. Studies in natural conditions are necessary to understand the factual role of spontaneous release of larvae and of “intermediesis” in the transmission of felid metastrongyloids to paratenic and definitive hosts.

Although it is a matter of fact that *T. brevior* is vertically transmitted, further data are warranted to elucidate how this occurs, i.e., in utero and/or lactogenically, and whether the vertical transmission takes place after recent infection in a late phase of pregnancy (as for the roundworm *T. cati* [74]), or in chronically infected queens that become pregnant. The hypothesized potential growth of *O. rostratus* larvae after hatching [34] should be further demonstrated. The description of the life cycle of *A. chabaudi* is partial and it is likely that it is impossible to complete because experimental infections and necropsy of the threatened natural host are unfeasible. Nonetheless, important information on the development of the nematode in intermediate hosts and on the involvement of mollusc species in its biology have been generated. The factual role of angiostrongylosis in cat parasitology (i.e., rare and negligible) has been elucidated, though undetected cases cannot be excluded given that infections are not patent and can be diagnosed only at necropsy or with purposed serological assays [36,37,62,63].

Knowledge of the identity of cardiopulmonary nematodes in wild felids is still meagre. Parasitological studies of elusive felids are difficult due the inherent hindrances in collecting suitable samples, but further studies are encouraged to implement knowledge of their cardiopulmonary parasitoses. Any opportunity should be approached in the best possible way to obtain conclusive results. As a recent example, genetic examinations and microscopic descriptions of parasites from kodkods in Chile would have allowed identification of *Troglostrongylus* sp. and *Angiostrongylus* sp. at the species level [64]. Furthermore, the species *A. felineus* has been described only microscopically, once, in a jaguarundi in Brazil [93], thus it is practically unknown and worth investigation. No molecular data are available, and it remains to be understood whether it is a valid species or a morphotype of *A. chabaudi*, and what are its geographical and host ranges. In general, it would be pivotal to investigate which nematodes actually affect elusive animals, and if felids living in remote geographies harbour unknown metastrongyloids. 

In conclusion, original research perspectives have been opened by recent knowledge and new questions are now posed. Therefore new studies will be conducted in the near future: it is likely that they will fill the gaps that have emerged and, at the same time, will pose further questions. The last few years have shown that DNA-based tools are powerful for the identification of metastrongyloids regardless of their biological stages. In fact, molecular tools have been successfully used in basic and applied studies in both intermediate and definitive hosts [7,8,15,17,19,25,37,38,57,58,63,69,77,98]. Therefore, they will be extremely useful in future studies on felid cardio-respiratory parasites.

## Figures and Tables

**Table 1:** Range of length (in µm) for the first stage larvae (L1) of *Aelurostrongylus abstrusus* and *Troglostrongylus brevior* reported in recent references.

*Aelurostrongylus abstrusus*	*Troglostrongylus brevior*	Ref.
300–400 ± 21.3	300–521	[3]
360–415 (399.1 ± 11.3)	300–357 (338.8 ± 15.6)	[9]
210.4–495.1	203.2–382.2	[16]

**Table 2:** Examples of descriptions (1977–2020) of *Aelurostrongylus* spp. in wild felids that could be attributed to other species of cardiopulmonary nematodes.

Felid	Measurements (µm) °	Identification	Feature Compatibility	Ref.
Cheetah (*Acinonyx jubatus*)	Not reported	*Aelurostrongylus abstrusus*	-	[39] *
Lion (*Panthera leo*)	Mean length ~280 ^#^ Mean width ~14 ^#^	*Aelurostrongylus abstrusus*	*Troglostrongylus* spp.	[40] *
Ocelot (*Leopardus pardalis*)	Not reported	*Aelurostrongylus abstrusus*	*-*	[41] **
Geoffroy’s cat (*Leopardus geoffroyi*)	Not reported	*Aelurostrongylus abstrusus*	*-*	[41] **
Eurasian lynx (*Lynx lynx*)	Length ~370-395 Width 18-25 ^#^	*Aelurostrongylus abstrusus*	*Angiostrongylus* spp./*Aelurostrongylus* spp.^@@^	[42] *
Leopard cat (*Prionailurus bengalensis*)	Length ~340-360 Width ~15 ^#^	*Aelurostrongylus abstrusus*	*Troglostrongylus* spp.	[43] *
Lion (*Panthera leo*)	Not reported ^@^	*Aelurostrongylus* spp.	-	[44]
Margay (*Leopardus wiedii*)	Not reported	*Aelurostrongylus* spp.	*-*	[45] **
Oncilla (*Leopardus tigrinus*)	Not reported	*Aelurostrongylus* spp.	*-*	[45] **
Lion (*Panthera leo*)	Length ~260 ^#^ Width 2.5 ^##^	*Aelurostrongylus* spp.	*Angiostrongylus* spp./*Aelurostrongylus* spp.^@@^	[46]
Amur tiger (*Panthera tigris altaica*)	Not reported	*Aelurostrongylus abstrusus*	*-*	[47]
Pampas cat (*Leopardus colocolo*)	Mean length 291±28 ^#^ Mean width 13±2 ^#^	*Aelurostrongylus abstrusus*	*Troglostrongylus* spp./*Angiostrongylus* spp./*Aelurostrongylus* spp.^§^	[48] **
Amur leopard (*Panthera pardus orientalis*)	Mean length 312 Mean width 17	*Troglostrongylus brevior*	*Angiostrongylus* spp./*Aelurostrongylus* spp.^@@^	[49]

° All identifications were based on first stage larvae (L1). In [39] adult stages were also examined, but no measurements were provided. The length of the L1 in this Table was examined using the precise ranges published in [9]. A recent ensuing study [16] demonstrated that the length ranges of *Aelurostrongylus abstrusus* and *Troglostrongylus brevior* L1 may be wider. * Cited in [9]; ** Cited in [50]; ^#^ The measurement is not compatible with *Aelurostrongylus abstrusus* according to the precise ranges published in [9]; ^##^ The width is out of any reported range for felid metastrongyloid larvae; ^§^ The quality of picture in the article does not allow us to draw any conclusion on the identity of the larva. The tail, blurred, looks like *Angiostrongylus* spp.; the length could be more compatible with *Troglostrongylus* spp. according to [9]; ^@^ Identification based on a kinked tail, insufficient for a definitive identification; ^@@^ The sigmoid tail with dorsal spine and notch, though blurred, looks like *Angiostrongylus* spp. or even *Aelurostrongylus* spp.

**Table 3:** Main cardio-respiratory metastrongyloid nematodes infecting domestic cats. Primary host, geographical distribution, clinical importance in *F. catus* and gaps of knowledge and open perspectives. * Recorded only in non-patent infections.

Parasite	Primary Host	Geographic Distribution	Clinical Importance	Fields to Investigate	Ref.
*Aelurostrongylus abstrusus*	*Felis catus*	Common Worldwide	Mild to severe Rarely fatal	Epizootiology in domestic and wild felids Alternative transmission patterns Diagnostic and control measures	[11,96,97,98]
*Troglostrongylus brevior*	*Felis silvestris*	Frequent Europe Middle East	Subclinical to mild in adults Severe and fatal in kittens	Epizootiology Alternative transmission patterns Vertical transmission Diagnostic and control measures	[11,12,14,98]
*Troglostrongylus subcrenatus*	Large wild felids ?	Extremely rare Africa Europe	Unknown Possibly fatal in kittens	Epizootiology Taxonomical status	[2]
*Oslerus rostratus*	Lynx ?	Sporadic Asia USA Europe	Negligible	Epizootiology Biology Clinical impact	[11,16,34]
*Angiostrongylus chabaudi*	*Felis silvestris*	Europe	Nil/Negligible *	Epizootiology Biology Clinical impact	[36,37,63]
*Angiostrongylus vasorum*	Canids	Worldwide	Nil/Negligible *	Epizootiology Biology Clinical impact	[50,62,99]

## Data Availability

The data presented in this study are available in all papers cited.
